# Challenges and opportunities of optimal breastfeeding in the context of HIV option B+ guidelines

**DOI:** 10.1186/s12889-017-4457-7

**Published:** 2017-06-02

**Authors:** Pamela Marinda, Nkandu Chibwe, Ernest Tambo, Sidney Lulanga, Christopher Khayeka—Wandabwa

**Affiliations:** 1 Department of Food Science and Nutrition, The University of Zambia, School of Agricultural Sciences, Lusaka, Zambia; 2 Choma District Hospital, Choma, Zambia; 3 Africa Disease Intelligence and Surveillance, Communication and Response (Africa DISCoR) Foundation, Yaoundé, Cameroon; 4 Department Biochemistry and Pharmaceutical Sciences, Higher Institute of Health Sciences, Université des Montagnes, Bangangté, Cameroon; 5 International University of Management, Faculty of Humanities, HIV/AIDS and Sustainable Development, 21-31 Hercules Street, Private Bag: 14005 Bachbrecht, Windhoek, Namibia; 6 African Population and Health Research Center (APHRC), P.O. Box 10787-00100, Nairobi, Kenya; 7 School of Pharmaceutical Science and Technology (SPST), Health Sciences Platform, Tianjin University, 300072 Tianjin, China

**Keywords:** Option B +, Human immunodeficiency virus (HIV), Antiretroviral drugs (ARVs), Antiretroviral therapy (ART), Prevention of mother to child transmission (PMTCT), Breastfeeding and pregnancy

## Abstract

**Background:**

In 2013, the World Health Organization released a new set of guidelines widely known as Option B+. Prior to that there were guidelines released in 2010. Option B+ recommends lifelong antiretroviral treatment for all pregnant and breastfeeding women living with Human Immunodeficiency Virus. The study aimed at investigating challenges and opportunities in implementing Infant and Young Child Feeding in the context of Prevention of Mother To Child Transmission (PMTCT) guidelines among HIV positive mothers of children aged 0–24 months. The study also examined implications presented by implementing the 2013 PMTCT consolidated guidelines in the transition phase from the 2010 approach in Zambia.

**Methods:**

A mixed methods approach was employed in the descriptive cross sectional study utilizing semi structured questionnaires and Focused Group Discussions. Further, data was captured from the Health Information Management System.

**Results:**

During the PMTCT transition, associated needs and challenges in institutionalizing the enhanced guidelines from option A and B to option B+ were observed. Nonetheless, there was a decline in Mother to Child Transmission (MTCT) of HIV rates with an average of 4%. Mothers faced challenges in complying with optimal breastfeeding practices owing to lack of community support systems and breast infections due to poor breast feeding occasioned by infants’ oral health challenges. Moreover, some mothers were hesitant of lifelong ARVs. Health workers faced programmatic and operational challenges such as compromised counseling services.

**Conclusion:**

Despite the ambitious timelines for PMTCT transition, the need to inculcate new knowledge and vary known practice among mothers and the shift in counseling content for health workers, the consolidated guidelines for PMTCT proved effective. Some mothers were hesitant of lifelong ARVs, rationalizing the debated paradigm that prolonged chemotherapy/polypharmacy may be a future challenge in the success of ART in PMTCT. Conflicting breast feeding practices was a common observation across mothers thus underpinning the need to strongly invigorate Infant and Young Child Feeding information sharing across the continuum of heath care from facility level to community and up to the family; for cultural norms, practices and attitudes enshrined within communities play a vital role in child care.

## Background

Optimal breastfeeding is essential for child survival and development because breast milk has all the necessary nutrients for healthy growth and provides significant protection from childhood diseases [1]. According to World Health Organization (WHO) recommendations for new born health; all babies should exclusively breastfeed from birth up to 6 months of age and their mothers counseled and given support for exclusive breastfeeding at each postnatal visit [2]. The child should be introduced to complementary foods at six months while continuing breastfeeding for up to 12 months if adequate complementary foods which are nutritious and safe can be sustained [2]. Such well-meaning guidelines when viewed in the framework of Prevention of Mother To Child Transmission (PMTCT) of HIV are not exempted from challenges [3, 4].

Considerable advances have been made in the effort towards preventing mother-to-child HIV transmission in sub-Saharan Africa, including clinical trials that have provided evidence on efficacy of antiretroviral regimes that can prevent HIV transmission during pregnancy, delivery and lactation period [3, 5, 6]. In 2013, WHO released a new set of guidelines dubbed Option B+ in which, as soon as diagnosed, all pregnant women living with HIV are offered life-long antiretroviral treatment, regardless of their cluster of differentiation four (CD4) count while the HIV exposed infants receive daily Nevirapine (NVP) or Azidothymidine (AZT) from birth up to six weeks regardless of infant feeding method [5, 7, 8]. Prior to the 2013 guidelines, there were the WHO 2010 PMTCT guidelines option A and option B for pregnant women living with HIV with CD4 greater than 350 cells/mm^3^ which Zambia had already adopted and was implementing. Overall, under Option A, mothers receive antepartum and intrapartum ARV prophylaxis along with postpartum regimen whereas the infants receive postpartum ARV prophylaxis throughout the duration of breastfeeding [7, 9]. Option B on the other hand entailed triple ARVs starting at 14 weeks of gestation and continued intrapartum and through childbirth if not breastfeeding or until 1 week after cessation of all breastfeeding whereas infants receive daily NVP or AZT from birth through age 4–6 weeks irrespective of infant feeding method utilized [7, 8]. At the close of breastfeeding, women who do not yet require ART would discontinue the prophylaxis and continue to monitor their CD4 count in the long run re-starting ART when the CD4 falls below 350 cells/mm [7].

In 2013, Zambia adopted the 2013 consolidated guidelines considered to be progressive from the 2010 recommendations [7, 8, 10]. The transition from 2010 course of action and subsequent adjustments in Infant and Young Child Feeding (IYCF) in tune with PMTCT strategies to fit the 2013 guidelines led to the need for the government to progressively revise health care providers’ PMTCT training packages. This need attracted extra costs and contributed to the staggered and delayed implementation. It equally occasioned a lapse in revision and harmonization of the IYCF training package for health care providers and inclusion of the same into community training package. Majority of resource-constrained countries in sub-Saharan Africa face related challenges in making adjustments to meet and implement the WHO guidelines due to human resource capacity, limiting educational strategies targeting Infant and Young Child Feeding and PMTCT policies [3, 4]. Several studies indicate that, changes in such or related critical guidelines have often contributed to low implementation rates. The low execution and efficiency is often attributed to staff adjustment constraints factoring already overburdened and limited human resource in the health sector, inconsistencies in information provided to mothers and therefore lack of adherence by mothers because of some level of confusion alongside adjustment lapse and unpredictable funds for ART 17,19 [11, 12].

Choma district in Zambia is one of the model districts that was chosen to implement Option B+ as an advancement from the 2010 guidelines. The changes occurred too fast before an evaluation of the 2010 PMTCT guidelines could be done at the facilities. The aim of the study was thus to investigate challenges and opportunities of implementing IYCF and PMTCT guidelines among HIV positive mothers of children 0–24 months and health care providers as well as examine implications presented by implementing the 2013 consolidated guidelines in Maternal and Child Health (MCH) in Choma district in Zambia. Like other developing nations, Zambia has a high number of people living with HIV/AIDS who are within the reproductive age bracket of 15–49 years old and are mainly women [13]. Global trends indicate that, mother to child transmission is estimated to account for over 90% of new HIV infection in children [14]. In absence of treatment, the likelihood of HIV passing from mother-to-child is considered to be between 15%–45% [6, 14] whereas, antiretroviral treatment and other effective PMTCT interventions can decrease this risk to below 5% as reported by WHO. Similarly, longitudinal surveys have emphasized progressive identification of pointers that predict PMTCT program performance thus gearing towards triggering timely identification of implementation problems and challenges that need corrective action for a more strengthened and progressive expansion of PMTCT services, particularly in sub-Saharan Africa [3, 15]. This is in tune with the United Nations Programme on HIV/AIDS (UNAID) and President’s Emergency Plan for AIDS Relief (PEPFAR) among other partners quest towards Global PMTCT targets. It also resonates with the recently launched Start Free, Stay Free, AIDS Free – a framework calling for a worldwide sprint towards “super fast-track targets”, to end AIDS among children, adolescents and young women by 2020. Hence, the need for incremental appraisal of evidence on cues to enhancing PMTCT in tandem with evolving policies, guidelines and practice.

## Methods

### Study setting

The study was conducted in Choma district which is situated in the Southern Province of Zambia along the rail line and the major Lusaka – Livingstone highway. It is 289 km (kms) south of Lusaka (the capital city of Zambia) and 188 kms north of Livingstone, one of the tourist cities in Zambia. According to the Choma District Community Medical Office action plan 2013, the expected pregnancies in the district were 5.4% (10812), expected deliveries, 5.2% (10411) and expected live births 4.95% (9911). Shampande and Railway Surgery clinics in Choma district were purposively selected for this study because they were among the model clinics in Zambia to start implementing the PMTCT program, had a high antenatal clinic case load and have a high burden of antenatal HIV (as reported in the District Community Medical Office Action plan, 2013).

### Study design

A cross sectional study design employing mixed methods approach was adopted in the survey. Semi structured questionnaires and focused group discussions (FGDs) were used to collect data. Data were collected between January and April 2015. The study population comprised of HIV positive mothers with children aged 0–24 months who attended PMTCT clinics, nurses who provide PMTCT services and had served in MCH departments for at least five months as well as community health volunteers.

### Quantitative methods

#### Sample size

Only mothers who had enrolled onto the PMTCT programme from January 2011 (date under the 2010 guidelines roll out) were considered for inclusion in the interviews. This was to ensure that we capture mothers who had transitioned from the 2010 to the 2013 WHO PMTCT guidelines adopted in Zambia. Therefore, a list with names of mothers who met the inclusion criteria in the selected facilities was compiled from the electronic register. Sample size for the mothers component was calculated based on the principle of single population proportion [16, 17]. Considering the proportion of expected mothers per Health Information Management System (HIMS) – Choma district, the estimated number of pregnancies in the catchment population was 5.4% of the total catchment population (Choma DCMO, HIMS, 2013). Hence **n = z** ^**2**^ **p (1-p)/ ∂** ^**2**^ where: n is the desired sample size; z represents the corresponding z score value at 95% confidence level (1.96); p is the estimated proportion of the target population of mothers enrolled into PMTCT program and ∂ is the margin of error. Therefore, *n* = 1.96^2^ *0.054(1–0.054) / 0.05^2^. Thus *n* = 79. To take care of attrition and non-response, a 6% attrition rate was expected and hence the sample size was adjusted upwards, to arrive at a study sample of eighty five (85). Thus, eighty five (85) mothers participated in the study. The mothers were stratified into two age group categories, those below and above 30 years old. Women in the two age categories may have different health seeking behaviors due to mainly differences in education, responsibilities and access to antenatal care [18–20], and may experience different challenges in IYCF. Simple random sampling was used to select participants from these two groups.

Ten (10) nurses (five from each clinic) were purposively sampled to participate in the study. Only those who had worked for a minimum of five months in the selected facilities and had been equally involved in transitional implementation of the new guidelines (either in the present facilities or related model facilities under government consideration) were interviewed on the IYCF/PMTCT services that were being provided at these two health facilities. Hence, the 10 nurses represent the total population of health personnel under the criteria providing MCH/PMTCT services at the facilities.

#### Data collection

A semi structured questionnaire for HIV positive mothers was used to collect information on maternal and infant demographic characteristics, maternal socio-economic characteristics, ANC/PMTCT services uptake and utility, the kind of challenges faced as regards breastfeeding and any social support received. Furthermore, health records on the two clinics were accessed from the Choma DCMO. Data on numbers of all mothers who underwent the PMTCT program, HIV positive mothers enrolled into the PMTCT programme, number of exposed infants taking HIV test, MTCT status from January 2011 to December 2014 was retrieved from these records.

Data on antenatal visits, institutional deliveries, HIV status of infants and mothers for the period 2011–2014, infant and young child feeding practices for select clinics was captured from the Health Information Management System (HIMS). An electronic system that tracks relevant clinical information for patients per clinical guidelines across the continuum of health care as earlier described [8, 13, 21–23]. The district health information system feeds into the nation health information system, via the provincial level system. Information from all health facilities within Choma is centrally available at the Choma district health information system unit. Health centres use duplicate systems i.e. paper based (patient cards, registers and tally sheets) and electronic record systems (smartcare) to record client information on a daily basis and ensure all records are progressively captured in electronic version. This is compiled at the end of the month for onward transmission to the district where it is aggregated for progressive submission. Before entries, compilation and aggregation are made and transmitted; reports are checked for completeness correctness and consistence at each level enhanced by systems quality control checks standards for quality threshold. The data was available in excel format and only information on variables of interest for the study was extracted and was found to be complete.

A self-administered semi structured questionnaire was used to collect data from health care providers on service delivery information which included: PMTCT/IYCF training, orientations and national guidelines/recommendations, ANC/PMTCT services provision, acceptance and challenges faced in provision of PMTCT/IYCF services.

#### Data analysis

Data collected from the interview and HIMS were entered and analyzed using Statistical Package for Social Sciences (SPSS) version 21. Quantitative data was analyzed by means of descriptive statistics.

### Qualitative methods

#### Selection of respondents

For focus groups discussion, participants were selected through a combination of convenience and purposeful sampling. Thus, for the pre-screening inclusion guide, the lead researchers agreed by consensus on an inclusion criterion that purposively targeted: (1) volunteers who had received training or orientation (in HIV testing, Infant and young child feeding counseling, Growth Monitoring and Promotion, Adherence counseling, PMTCT lay counseling and related guidelines) and were actively serving the selected facilities MCH departments in tune with Infant and Young Child Feeding (IYCF) as well as PMTCT guidelines, (2) Volunteers who had worked consistently for a minimum of five months in the selected facilities at the time of the study and had prior involvement in transitional implementation of the new PMTCT guidelines. Verification of CHVs meeting the criteria was based on stakeholder recommendations informed by Zambia’s National Community Health Worker Strategy unit on training and tracking of CHVs involvement in the health systems and services delivery [24]. As a result, from the pre-screened CHVs list as guided by inclusion criteria, a sample was purposively selected factoring convenience. Consequently, six CHVs were available for the FGD at Shampande and six from Railway attended out of the targeted eight hence, the considered final coverage from both facilities was deemed sufficient for FGDs scope of objective [25, 26] incorporating the healthcare providers who were willing and available.

#### Data collection

The two FGDs, one per site were aimed at eliciting information on kinds of community support systems provided to breastfeeding mothers in general and to HIV positive mothers, any challenges faced in PMTCT/IYCF activities and breastfeeding practices in order to cross check mothers responses. The conduct and reporting presented in this paper adhered to the consolidated criteria for reporting qualitative research (COREQ) guidelines [27]. A qualitative design using focus group discussion (FGD) methodology was applied as is an ideal approach to explore perceived motivators and barriers to healthy behaviors [28]. The Focus group discussion sessions lasted between 43 to 65 min. In the course of data collection, probes were used to clarify, and explore the topics. In the concluding minutes of each FGD session a verbal summary of responses was provided to participants by the moderator. Participants were asked to review the summary with an option to provide any other additional comments that may have been missed. The items in FGD interviews were initially formulated in English then translated into *Tonga*, (a language that is commonly spoken in Southern province of Zambia) for the use in the study location. Data collected in *Tonga* versions were translated back to English to ensure consistency with the data collected in English versions. All data tools used were pretested at a non-participating Choma Hospital Affiliated Health Centre (HAHC) where HIV positive mothers and health care providers could be accessed for the pretest exercise.

#### Data analysis

All the data from the FGDs; supported by the comprehensive handwritten field notes were transcribed and analyzed into themes manually using the principles of systematic text condensation as described by Malterud [29, 30].The qualitative data generated was organized into thematic content which involved identifying main categories and recurrent themes concerning challenges and opportunities to optimal infant feeding in the context of PMTCT.

## Results

### Demographic characteristics

The study population involved a total of 85 HIV positive breastfeeding mothers aged between 16 and 42 years who were categorized into two age groups in years (30 < age ≤ 30) with an overall mean age of 28 years (Table 1). Pearson’s chi-squared test was applied to establish whether there was a significant difference between level of education of mothers and their ability to recite health education and counseling information received during clinic visits. From the evaluation, there was no statistically significant difference in the level of education of mothers and the ability to recite the information given during health education sessions and counseling (*p* = 0.479).

**Table 1:** Demographic and socio economic characteristics of respondents included in this study (*N* = 85)

Characteristics	N (%)
Childs’ Age	
0–6 months	38 (45%)
7–11 months	25 (29%)
12–24 months	22 (26%)
Mothers’ Age	
Below 30	53 (62%)
Above 30	32 (38%)
Marital status	
Single	17 (20%)
Married	62 (73%)
Divorced	3 (4%)
Widowed	2 (2%)
Cohabiting	1 (1%)
Mothers’ occupation	
Formal	7 (8%)
Informal	12 (14%)
None	66 (78%)
Educational level	
Primary	25 (29%)
Secondary	49 (58%)
Tertiary	10 (12%)
N/A	1 (1%)
Fathers occupation	
Formal	27 (32%)
Informal	38 (45%)
None	18 (21%)
N/A	2 (2%)

*N/A* Not applicable

Healthcare providers from the selected study setting who provide MCH services and had served for more than five months in MCH were interviewed alongside conducting two focused group discussions with volunteers from respective clinics. The kinds of community support systems provided to breastfeeding mothers in general and to HIV positive mothers, any challenges faced in PMTCT/IYCF activities and breastfeeding practices reporting in relation to option B+ ART was tackled based on three core topical areas that emerged: (a) opportunities for optimal breastfeeding in the context of HIV Option B+ guidelines, (b) opportunities for promotion of optimal breastfeeding and support for HIV positive mothers and (c) programmatic and operational challenges. Further key sub-thematic points are elaborated under the core topical areas (b and c) as elaborated alongside triangulation of qualitative and quantitative data collected.

### Opportunities for optimal breastfeeding in the context of HIV option B+ guidelines

**Fig. 1:**
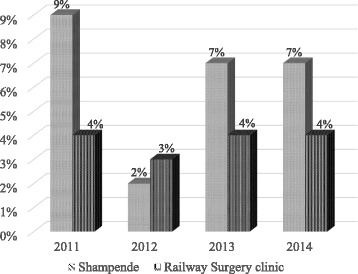
Proportion of HIV positive infants born from HIV positive mothers between 2011 and 2014

Data from the HIMS was used to determine the proportion of HIV+ infants born from HIV+ mothers between 2011 and 2014 for Shampande and Railway Surgery clinics of Choma district in Zambia (Fig. 1). Results reveal a decrease in mother to child transmission of HIV at both clinics from 2011 to 2012 by *7*% and 1%, respectively. In 2013, records showed an increase in MTCT at both clinics by 5% and 1%. From 2013 to 2014, there was no change in MTCT at both clinics*.* According to the health care providers, observed findings were attributed to the changes that occurred in 2013. A time when WHO revised the PMTCT guidelines and recommended that in low-and middle-income countries option B+ for treatment of HIV infected mothers be followed and in countries where this was not feasible, option B be adopted. The nurses indicated that, during the transition from option B to option B+ mothers got confused with regard to the changes and practiced mixed feeding during that phase. They further specified that some mothers were not ready to take ARVs for life and absconded.

*“Some mothers are reluctant and do not want to start ART for life but would collect drugs for their babies” (Nurse counselor # 7).*

It also took long before a good number of health workers underwent training on the new guidelines (option B+) while others only received an orientation on the changes. Due to health education during the transition, mothers have come to understand the changes including the benefits therewith and are taking ARVs with minimal challenges.

### Opportunities for promotion of optimal breastfeeding and support for HIV positive mothers

#### Focused antenatal care

**Fig. 2:**
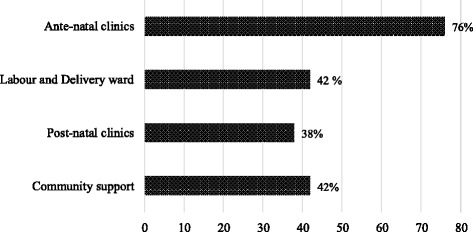
Proportion of mothers receiving support information on breastfeeding and IYCF from various sources

Findings from the two clinics indicated that about 59% of the mothers (within the 4 years period 2011–2014) on average began their ANC after 20 weeks gestation. Over three quarters of the HIV positive mothers reported to have received information on exclusive breast feeding while attending antenatal care. The information source was majorly from health care providers and fellow mothers during their interaction. Other sources of information included during postnatal clinic attendance and from the community as presented in Fig. 2.

#### Infant and young child feeding

Majority of the mothers practiced breastfeeding (Table 2). At six months infants age, approximately a half of the mothers reported to have introduced complementary feeds whereas a small proportion introduced before six months. Beyond six months of infants age, majority of the mothers continued breastfeeding while only 21% stopped giving the breast milk. It is important to note, with respect to the trends observed, the number of visits that a mother makes to the clinic determines how much information she receives. Health education is given at each visit on different topics and this has an impact on the practices mothers have towards breastfeeding and the challenges that they may be facing.

**Table 2:** Mothers breastfeeding practices after delivery (*N* = 85)

Practices	N (%)
Method of feeding the Infant	
Breastfeeding	83 (98%)
Replacement feeding	2 (2%)
Age when complementary foods were introduced	
Before 6 months	5 (6%)
After 6 months	43 (51%)
EBF	37 (43%)
Continued breastfeeding	
Yes	65 (77%)
No	18 (21%)
N/A	2 (2%)

*N/A* Not applicable, *EBF* Exclusive Breastfeeding

#### Male involvement, community and family support systems

Focused group discussions with PMTCT/IYCF community volunteers’ counselors indicated that, there exist support systems within the community for mothers for infant feeding practices among family members, friends and volunteers. These entailed an empowered community environment with members (majorly community health volunteers) capable of supporting timely service uptake for all women through peer mother groups and male partner involvement. Through this, HIV-positive pregnant women are encouraged on a number of good health practices such as timely initiation of treatment and adherence to instructions on taking of ART. Thus, expert patients (in this case, HIV positive mothers who have experience) as well as mother groups, and community health volunteers are engaged in providing support to HIV positive mothers for enhanced ANC. Information provided to the mothers and shared across include: proper positioning and attachment of baby on the breast, breast feeding on demand, to recognize danger signs on their breasts and prevent HIV transmission and exclusive breast feeding for 6 months. The observations concurs with what was captured from mothers interviews; which indicated that 62% (*n* = 44) of the mothers had received some form of support for infant feeding via community support channels predominantly from the community health volunteers but also empowered peer mother groups.

Some of the volunteers at the clinic were male and they used their position to encourage the men in the community to support their spouses. Male partners’ involvement was in the form of providing support to their wives by bringing their children for health care services (collection of drugs and under five counselling) and receiving information on reproductive health. The clinics had set aside a separate day for men to bring their children for services. One volunteer was quoted as follows:

*‘We have a specific day (on Tuesdays) when fathers are allowed to collect drugs for their children and on under five days they are given first priority” (CHV # 2).*

Of the 85 mothers attending antenatal care, 71% (*n* = 60) were accompanied by their partners to the antenatal clinic while 28% (*n* = 24) attended unaccompanied.

#### Health education and clinic days

Health education and counseling is given at each clinic visit on varied topics. This is done to raise awareness on different issues including clarifying questions that arise during discussions. This is important as seen from the results that mothers were able to recite what they learnt regardless of education status. A separate day/s is set aside for provision of PMTCT services for HIV + mothers and the exposed infants at both Shampande and Railway Surgery clinics. The staff mentioned that they were prompted to do so because of the difficulties they faced in tracking the Mother/baby pairs for provision of PMTCT services and the increase in the loss to follow clients. During the clinics, mothers have an opportunity to discuss HIV issues among themselves promoting expert patients way of information sharing. It has also made it easier for nurses to avail PMTCT service with more ease and capture data for HIV positive mothers in a timely manner and with enhanced precision as noted in the interviews:

“*It has now become very easy for us to deal with them (HIV infected mothers) separately because we talk about issues that concern all of them unlike before when we used to mix them with the rest of the other mothers. They even discuss HIV issues amongst themselves” (CHV # 1).*

*“Work has been made easier especially when capturing data in the registers, because you are dealing with clients of similar conditions. It is even faster to provide the service unlike previously when we used to identify them (HIV+ mothers) from the crowd, we could even miss them” (Nurse counselor # 1).*

#### Tapping on skilled volunteers capability

Both clinics have several volunteers (with a technical training) from the surrounding communities who come through to the clinic on different days to assist in conducting HIV testing, providing counseling, weighing the children, withdrawing blood for Dry Blood Spot among other activities. From the presented context regarding IYFC and PMTCT trends in the two clinics of Choma district, it is evident that, “PMTCT cascade” represents the critical pathway pregnant women and their infants must successfully go through to benefit fully from PMTCT services. These steps are not limited to but include women testing for HIV, receiving their results, undergoing ART eligibility screening, initiating treatment, and adhering to the prescribed regimens. Majority of nurses pointed that mothers would easily be linked to known volunteer serving the facility but residing within the communities for any pressing support information that may not necessarily need a facility visit. This was viewed as an innovative dimension by health workers in supporting the women in a setting where transport and communication has a socio economic bearing and burden. The facilities also end up having some ample working framework to attend to programmatic core services on scheduled clinic visit days.

### Challenges to optimal breast feeding and ARVs

It was observed that, 15% (*n* = 13) of the mothers who participated in the study reported to have had some breastfeeding problem at some point during the breastfeeding period. The problems ranged from breast conditions such as engorged breasts, inadequate milk, cracked nipples or breast sores. Thirty two percent (32%; *n* = 27) of the mothers reported to have had difficult experiences as HIV positive mothers in their breastfeeding period which included; stigma, fear of transmitting the virus to their babies, oral thrush in baby’s mouth, lack of support from the family, developing breast conditions because of the baby’s inability to breast feed due to oral thrush in the baby’s mouth, mother returning to work and sustaining breastfeeding. About 48% (*n* = 41) of the mothers reported not to have received any support in their breastfeeding experience while the 68% (*n* = 44) had received some form of support, which included help on position and attachment of the baby to the breast, how to sustain exclusive breastfeeding, and how to express milk for the baby when separated from them. However, at the time of data collection Nevirapine for infants was out of stock thus, mothers were afraid that they would transmit the HIV virus to their infants.

### Programmatic and operational challenges

#### Service delivery

The challenges faced by healthcare providers in service delivery are presented in Table 3. They were grouped into three main domains: challenges related to counselling services, supplies and workload.

**Table 3:** Challenges faced by health care providers in service delivery

Group	Sub-group
Counseling	Inadequate space for privacy, all the services provided in the same place Same nurse provides all the services during their shift Few volunteers to help out Many registers for recording the data collected
Supplies	No Nevirapine No Dried Blood Spots kits Many mothers seek the service compared to capacity Working mothers and school going adolescents do not come to get supplies for their children Some mothers refuse to get treatment for themselves
Work flow management	Too much work, too few staff Integration of ART in MCH overwhelming

#### Counseling, multi-tasking, time constraint within shifts and integration of ART in MCH Department

The health care providers interviewed indicated that there was inadequate space for conducting counseling in privacy for all mothers. The same space is used as labour ward, houses postnatal mothers for 6 h after delivery prior to discharge as well as other MCH activities such as growth monitoring and promotion and family planning. They also reported that before integrating ART in MCH services, there was enough space to provide these services. With the Option B+ strategy, the health care providers are expected to provide all the services under one umbrella at both clinics. This had caused a lapse in the quality of counseling provided as there was inadequate space and privacy to conduct the counseling as widely observed by the nurses interviewed:

*“You can imagine that we provide all the services in this same room, despite the integration of ART in MCH for pregnant mothers, nothing has been done on the infrastructure” (Nurse counselor # 2).*

*“Can you imagine you are counseling a woman on adherence and an ANC mother is being dealt with by another nurse in this same place” (Nurse counselor # 3).*

Multi-tasking and time was a constraint for both the nurses who provided counselling services and the mothers who had to cue for long. Nurses interviewed reported to be overwhelmed by the high demand for counselling services resulting to compromise on quality:

*“Imagine if I have to counsel the same woman on adherence, infant feeding and family planning, so you leave out some information or just scratch on the surface to give chance to everyone who has attended the clinic” (Nurse counsellor # 4).*

Due to insufficient counseling services provided, some mothers who do not fully understand the importance of the strategy tend to abscond. It results to others delaying to make the decision to start receiving treatment but would in the meantime allow their babies to receive the drugs. Working class mothers and mother attending schools/colleges are unable to bring their children for the services regularly due to uncertainty of lapse time before getting real services. However, they do send caretakers to bring their children. With regard to work load, some observation were noted:

*“For example during my shift, as you have seen, I have a woman in labour and all these women you see have to be counseled after posttest if it’s an antenatal booking day; and I have to dispense the ARVs and other things like entering in the register. Also on a day like this, for those (mothers) beginning they need to be counseled on adherence as a requirement for ART”. (Nurse counselor # 5).*

*“Once I sit here when I report for work, it’s until the next person on duty reports and I don’t leave the seat. You make sure you eat before coming for work or else you don’t find time to put something in your mouth” (Nurse counselor # 6).*

Alongside the manual (paper based) record entry, there is an electronic record system known as Smartcare which is used by the two clinics. Ideally, all the data should be captured and entered in the same manner in both the paper-based register and electronic system and the combined records mode increases the workload. The practice is to use the paper based system first, then later all the data from the paper based register is entered in the electronic system before the end of the day. Despite efforts to ensure correct data entry in both systems, the reality is that there exist gaps in these registers because of the numerous records that need to be updated on regular basis with information collected. The records to be updated include the ART file, drug log book, Dried Blood Spot (DBS) tracking, PMCT register, HIV daily activity log book, tally sheets among others. A nurse was quoted saying:

*“For example there is the ART file to update, drug log book, DBS tracking, PMTCT register, HIV daily activity log book, tally sheets…. All these need to be updated with the information collected” (Nurse counselor # 2).*

Integration and management of ART in MCH department has come with its own challenges as aforementioned and further shared by the health service providers interviewed:

*“That’s why we set aside a specific day for PMTCT mothers to come for their supplies and DBS collection so that it’s easy for us to provide the service specifically for them unlike when they used to come for under 5 clinic with the rest of the mothers” (Nurse counselor # 6).*

#### Supplies

The health care providers expressed their concern on the challenge of inadequate supplies. The staff confirmed that since mothers have become more aware of the importance of PMTCT, many come to the clinics as required and as such, there is increased demand for supplies such as ARV drugs, HIV testing kits, DBS kits, and antibiotics. Therefore, these supplies tend to run out:

*“Currently the Nevirapine and DBS kits have been out of stock for some time now, the Pharmacist called this morning that he has a few bottles he has to share among the few busy clinics and so we must place an order for Nevirapine” (Nurse counselor # 7).*

*“We have not been collecting DBS sample for 3 months now and mothers are anxious to know their children’s status” (Nurse counselor # 8).*

## Discussion

From the presented findings, the overall children’s age ranged from 0 to 24 months and mean age was eight months. Of the 85 mothers who participated, 20% were single mothers and mostly school going adolescents. The mothers interviewed demonstrated high levels of knowledge (60%) on what they learnt during clinic visits. The consolidated guidelines for PMTCT proved effective despite the inherent health systems challenges alongside innovative approaches and commitments by both the health providers and the community (at family or volunteers level) to ensure the success of the intended aims of PMTCT programme. The Zambia government has put in place a system where adolescents who fall pregnant are allowed to attend school under special arrangement (i.e. afternoon school/evening school). Adolescent pregnancies in this study underpin the increasing double challenge trend in sub-Saharan Africa of early pregnancy and increased HIV among young adults and school going age teenagers. Thus, the increasing need for locally adaptable strategies [13, 31]. The proportion of mothers who had attained secondary and primary level education emphasizes the need for simplified packaging of educational materials for increased impact of PMTCT and related programmes in resource limited settings [32]. The indicators further demonstrate the importance of addressing individual-level factors (level of education, livelihood determinants and family support among others) through education and counseling in medical interventions for PMTCT for the mothers during their clinical visits. It is possible, context specific targeted efforts (such as health education mode of delivery through special clinic days and male partner involvement) to improve adherence to recommended PMTCT guidelines by health care providers to all eligible mothers played a key role [33, 34] in the mothers level of knowledge observed. Enhanced levels of knowledge by mothers has been shown to be instrumental in aiding them understand health seeking behaviours and enable them to abide to counseling messages obtained from the clinic [35].

As earlier pointed, mother to child transmission is estimated to account for over 90% of new HIV infection in children and in absence of treatment, the likelihood of HIV passing from mother-to-child is considered to be between 15%–45% [6, 14, 36]. Use of antiretroviral treatment and other effective PMTCT interventions can decrease this risk to below 5% as reported by WHO. Earlier studies have also demonstrated the association between positivity rates among HIV-exposed infants with changes in prevention of mother-to-child transmission efforts. The association has been in the dimension of mother-child pairs neither receiving ARVs, mixed feeding practices or mothers not adhering to institutional ANC and postnatal care across the continuum of health care [37]. Thus, the findings on proportion of HIV positive infants born from HIV positive mothers between 2011 and 2014 point to the long debated paradigm that; the success of ART in PMTCT and improving maternal treatment is constrained by knowledge gaps about optimal maternal regimens, duration of infant prophylaxis alongside the short-term and long-term effects on both the mother and children. With the evidence surrounding PMTCT regimens constantly evolving, the trend in MTCT in this study is in tune with most countries highly affected by HIV trying to ensure that their PMTCT programmes are effective. A 2012 snapshot of PMTCT regimens approved by ministries of health in twenty two countries experiencing a high burden of HIV infection revealed that almost a half of these countries had an Option A regimen policy in place although three of them had piloted Option B+ in selected settings; six had an Option B policy; and six had an Option B+ policy and reported varying decrease in MTCT rates from 26% to 17% in 2012 [38, 39]. An anticipated further decline in MTCT with the introduction of Option B+ and its subsequent full implementation were observed 50 [38]. The trend in challenges and opportunities of IYCF to PMTCT of HIV are in concomitant with earlier established observations [40, 41].

Presenting early at ANC is crucial for PMTCT so that interventions are made early. The findings reveal that most of the information is received at antenatal clinics and immediately after delivery. The observations underpin the relevance of supporting the integration of PMTCT and pediatric HIV with Maternal, Neonatal, and Child Health (MNCH) services at the level of policy, program administration and service delivery. This will provide an opportunity for leveraging on other key programs in scenarios of constrained health systems in developing countries like Zambia and many more in sub-Saharan Africa [5, 6]. The trend on sources of health information equally provides real world indication to the potential of community health volunteers/workers as a cornerstone workforce for the scaling up of community health delivery. The personnel would be core in ART with reporting lines, training, supervision, acceptability, expanded access to care and feedback in a bidirectional manner [42–44].

Exclusive breast feeding practices of majority of mothers were in line with the WHO (2012) recommendation on exclusive breast feeding. WHO aims at supporting countries with implementation and monitoring of the "Comprehensive implementation plan on maternal, infant and young child nutrition" through increasing the rate of exclusive breastfeeding for the first six months up to at least 50% by 2025 [2, 45]. For the first six months exclusive breast-feeding by HIV positive mothers in countries where replacement feeding is generally not affordable feasible, acceptable, sustainable or safe (AFASS) would be integral in the context of PMTCT [15, 46] which is the case in Zambia. In Zambia, before introduction of Option B+, HIV+ mothers could choose to breast feed or not to breastfeed. Introduction of Option B+ accompanied by the nutrition education support, has made it possible for more HIV+ mothers to breastfeed their infants. Findings from Botswana have demonstrated that the risk of MTCT with exclusive breast feeding is low when breast milk has low viral load which comes with ART [47].

Cultural norms, practices and attitudes that are enshrined within communities play an important role in child care practices, family support for HIV infected women and health promotion interventions [48–50]. Male partner and male volunteer involvement in IYCF/PMTCT was evident. Focused group discussions with PMTCT/IYCF community volunteers’ counselors indicated that, there exist support systems within the community for mothers for infant feeding practices among family members, friends and volunteers. The observations concurs with what was captured from mothers interviews; which indicated that 62% of the mothers had received some form of support for infant feeding via community support channels predominantly from the community health volunteers but also empowered peer mother groups. It is therefore eminent, as ambitious strategies and policies are established by governments and unprecedented resources deployed towards the fight against mother-to-child transmission of HIV across the globe, there is clearly a need to develop effective family-oriented and culture-centered community-based PMTCT interventions that could in the long run achieve comprehensive four pronged strategy of: (a) primary prevention and control of HIV infection among women of reproductive age, (b) the prevention of unplanned pregnancies among HIV positive women, (c) the prevention of HIV transmission from HIV infected mothers to their infants, and (d) the provision of care, support and empowerment for HIV infected mothers, infants and family members. When there is family support, there exists a huge potential for improving the effectiveness of PMTCT among HIV positive mothers [50]. The importance of male involvement cannot be underestimated in PMTCT programmes as they have important roles to play in care and maintenance of expectant mothers [51]. Male involvement increases uptake of reproductive choices through improvement of communication by spouses through pathways of increased knowledge or decreased male opposition [52].

When the infants are born, they also must adhere to antiretroviral prophylaxis regimens and undergo appropriately timed HIV testing and if they are found to be infected must initiate ART treatment timely. If there is attrition at any point in the chain, the system is seen as inefficient and limits program impact, reduces overall coverage, and leads to more infant HIV infection [4]. According to nurses interviewed, the unique model of involving skilled volunteers and male partners emanating from the communities within which the facilities operate has contributed to enhanced community-facility counter referral support mechanism. PATHFINDER global PMTCT strategy emphasizes the need to create an enabling environment within communities where members can provide PMTCT services to affected women within their communities [53, 54]. This is because social norms, stigma, gender-based inequities and socioeconomic factors influence the ability of women to practice what they learn at the clinic. In addition, poor infant feeding practices in the general population and increased rates of non-adherence to ART regimens are evidence of a poor enabling environment and the challenge associated with changing behaviours [41]. A referral linkage between facility services and community care is also crucial to avoid gaps in support and follow-up of these mothers if the PMTCT programme is to prove effective. To protect and improve health, especially in poor communities, combining community and facility based activities is required, which should be supported also by the national level policy and strategies.

From the study findings, all the mothers interviewed were on appropriate ARVs regimen despite the challenge of unsteady supply. Unsteady supply and availability of essential PMTCT commodities are among gaps that desire streamlining by many African governments health authorities [41, 47]. It is critical that, mothers take ARVs for their own health, all through the duration of breastfeeding to reduce viral load while their infants should receive ARVs for 4–6 weeks after birth in an effort to prevent postnatal vertical transmission of HIV [41, 47]. PMTCT interventions can be effective and highly successful in reducing the risk of HIV transmission especially in sub-Saharan Africa [40]. However, its efficiency is hampered by a lot of challenges including shortage of personnel, poor infrastructure and inadequate supply of PMTCT kits as well as other social cultural factors such as preference for home delivery. These factors as replicated in the present study, end up playing a key role in lack of effectiveness of PMTCT services. Even so, in order to provide services for more women in need of timely intervention, there is an increasing local and international support for integrating PMCT into MCH infrastructure [55] without further expansion to infrastructure being considered thus, compromising quality of services provided. Therefore, caution needs to be taken in ensuring integration of PMTCT in the overall MCH conforms to capacity needs in terms of infrastructure and human resource with proper monitoring and evaluation [55].

The conditions in Zambia are not unique, as other studies conducted in Tanzania and Uganda indicate that insufficient PMTCT information and counselling were provided by nurses due to time and human resource constraints [56, 57]. In Ethiopia, an increase in health providers’ workload with the introduction of the PMTCT services was observed [58]. Although community health volunteers/workers play an important role in the implementation of PMTCT, they can only play a complementary role. They cannot be key in overcoming the structural shortfalls in service provision in the regular health care systems as is the case in Zambia and prior findings in Uganda and Tanzania [56–58]***.*** With close to a decade of rigorous PMCT program advocacy, implementation and well known benefits, the aforementioned shared and unwavering challenges especially in sub- Saharan Africa warrants the need for opening up comprehensive discussion. Open debate and consultations among enthusiasts and cautious critics of option B+ [49] will potentially enable define better implementation approaches that are locally adaptable and pragmatic based on regional programmatic and economic concerns [15]. In linkage to the multi-record data capture and storage systems, emphasize the need to leverage on Smart-Care in the context of Zambia health system by enhancing its functionalities for efficiency [23]. Smart-care is the largest electronic medical record that was developed by the Ministry of Health in Zambia and Centre for Disease Control. It aimed at linking services for HIV patients while improving access to health information regardless of location, thereby mitigating delays in initiation of treatment, duplication of investigations, reduce risks and errors, expenses and improve HIV data standards, security and confidentiality [23]. The smart-care platform contains electronic forms that health providers use to record patient information that include counselling and testing, initial history and physical examination, investigations, medication and long term follow up. After entry of all the information, the data is copied to a smart card that has a unique pin number. In the two clinics where the study was conducted, there is duplication of effort, where both the paper based and electronic systems are used. Frequent electricity outages, breakdown of computers and inadequate IT support were reasons given for the need for a back-up manual paper system.

Despite the challenges integration of ART in MCH remains critical to the success in implementing options B+ in pre-natal and post-natal care. Especially through the breastfeeding period as opposed to referral of pregnant women and their babies to a separate ART clinic as was the case in Zambia previously. Due to integration, fewer mothers are lost to follow-up [6, 55]. Operational considerations indicate that incorporation of ART in MCH department ensures improved outcomes for HIV infected pregnant mothers and HIV-exposed children [6]. Experience from a Malawi programme indicated that ART initiation within MCH sites improved retention compared to referral to ART sites. However, the presented findings point to the fact that for the progressive success of merging PMTCT and MCH programs, such efforts should fit into good planning and allocation of human resources for health. In resource-poor settings, shortages of PMTCT staff, interruptions in treatment and supplies of medical equipment, as well as a shortfall in counseling services, all act as barriers to PMTCT services [32, 59]. These factors often mean long waiting times for post-test counseling.

In order to better understand the downstream impact of the Option B+ approach, more such “real world” program data are needed in sub-Saharan Africa and must be carefully evaluated. This means that the aggregate tallies made routinely in the HIMS, longitudinal patient-level data should be collected at representative sites, ideally linked between programs i.e. PMTCT, ART and between mother, child and community model partnerships to inform enhanced and well integrated health services delivery. Moreover, future insights in PMTCT counselling should also target women’s broader living conditions, that is economic factors and community-level factors (particularly stigma, fear of disclosure and partner support) [32]. Only through a combination of individual, community and structural interventions will we achieve an AIDS-free generation, which requires the elimination of vertical transmission of HIV in sub-Saharan Africa [32, 60]. The presented findings had some limitations. The research only looked at breastfeeding practices among HIV positive mothers and did not include complementary feeding part of the IYCF guidelines.

## Conclusion

Despite the ambitious timelines for PMTCT transition in the current study setting, the need to inculcate new knowledge and vary known practice among mothers due to prior guidelines and the shift in counseling content for health workers delivering maternal child health services, the consolidated guidelines for PMTCT were effective. Some notable best practice identified was importance of specific day(s) for health education to HIV positive mothers and their male partners’ involvement in the same as a way of community support at family level. Despite the underlying challenges, use of the Smartcare data capturing system enabled easy patient tracking. The fact that some mothers were not ready to take ARVs for life and absconded but adhered to desired infant care, rationalizes the long debated paradigm that prolonged chemotherapy and/or increasing trend of polypharmacy may be a future challenge in the success of ART in PMTCT. Conflicting breast feeding practices was a common observation across mothers thus underpinning the need to strongly invigorate Infant and Young Child Feeding (IYCF) information sharing across the continuum of heath care from facility level to community and upto family. Such efforts would be of immense benefit for cultural norms, practices and attitudes enshrined within communities play a vital role in child care. Longstanding deterrents; late presentation of mothers to antenatal clinics, anxiety combined with fear by mothers of infecting their babies with HIV due to prolonged breastfeeding and inadequate supplies are factors that continue to hinder the success of the Option B+ in PMTCT. Hence, a call for continued support and strengthening of counseling services not just in quantity but quality as well.

## Abbreviations

AFASS: Affordable feasible, acceptable, sustainable or safe
ANC: Antenatal care
ART: antiretroviral therapy
ARVs: Antiretroviral drugs
AZT: Azidothymidine
CD4: Cluster of differentiation four
CHVs: Community health volunteers
COREQ: Consolidated criteria for reporting qualitative research
DBS: Dried blood spot
DMCO: District community medical office
FGDs: Focused group discussions
HAHC: Hospital affiliated health centre
HIMS: Health information management system
HIV: Human immunodeficiency virus
IT: Information technology
IYCF: Infant and young child feeding
MCH: Maternal and child health
MTCT: Mother to child transmission
NVP: Nevirapine
PEPFAR: President’s emergency plan for AIDS relief
PMTCT: Prevention of mother to child transmission
SPSS: Statistical package for social sciences
UNAID: United Nations programme on HIV/AIDS
WHO: World health organization
